# Supplementary material for: A draft genome of Drung cattle reveals clues to its chromosomal fusion and environmental adaptation
Source: Commun Biol. 2022 Apr 13;5:353. doi: 10.1038/s42003-022-03298-9 (PMC9008013; doi:10.1038/s42003-022-03298-9)
Supplement: Supplementary file 2 — Supplementary Information [file 42003_2022_3298_MOESM2_ESM.pdf]

Supplementary materials

Supplementary figures

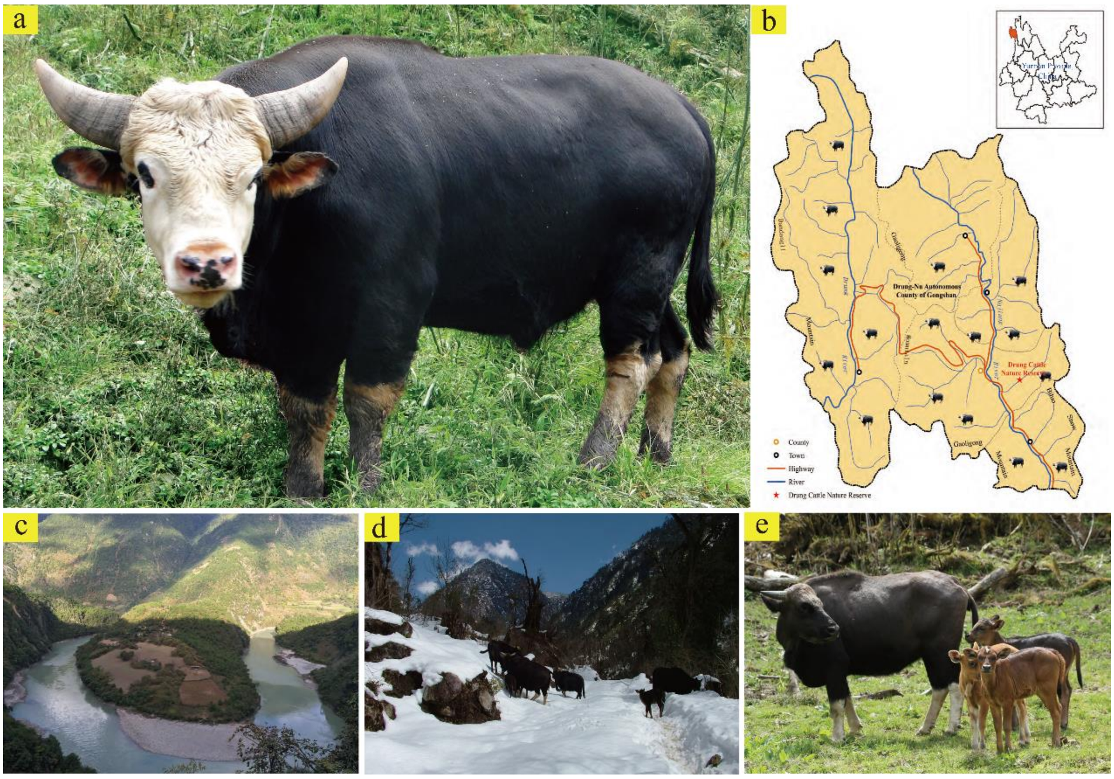

Supplementary Figure 1. The Drung cattle (*Bos frontalis*).

(a) Photograph. Showing typical characteristics of a wild adult Drung bull, white head and white stocking on four legs, with strong upper limbs and good trunk muscles. **(b)** Distribution. Drung cattle is primarily distributed in the Drung and Nujiang river basins, Northwestern Yunnan Province, China. Drung Cattle Nature Reserve has been established to protect and improve its ecological environment in the Drung-Nu Autonomous County of Gongshan. **(c)** Habitat. It is a gathering place for ethnic minorities such as Nu, Lisu, Dulong and Tibetan. Surrounded by high mountains, deep canyons, waterfalls, and river, this area is a subtropical plateau climate in complex terrain of a typical alpine gorge, at an average altitude of 3500 m above sea level. **(d)** Habits. Drung cattle is social animal, frequently congregating in groups and licking salt. It has strong foraging ability, even in the snowy winter, and the diet includes grass, leaves, twigs, bark, and herbaceous plants, preferring bamboo leaves and shoots. **(e)** Reproduction and Population. Males reach sexual maturity on average 4 years old, relatively later than *Bos taurus*. Females become capable of reproduction when at least

2-3 years old. The gestation period lasts 290-300 days. Drung cattle tend to live in herds led by a larger adult female, with 10-30 heads consisting of females, juveniles and subadults.

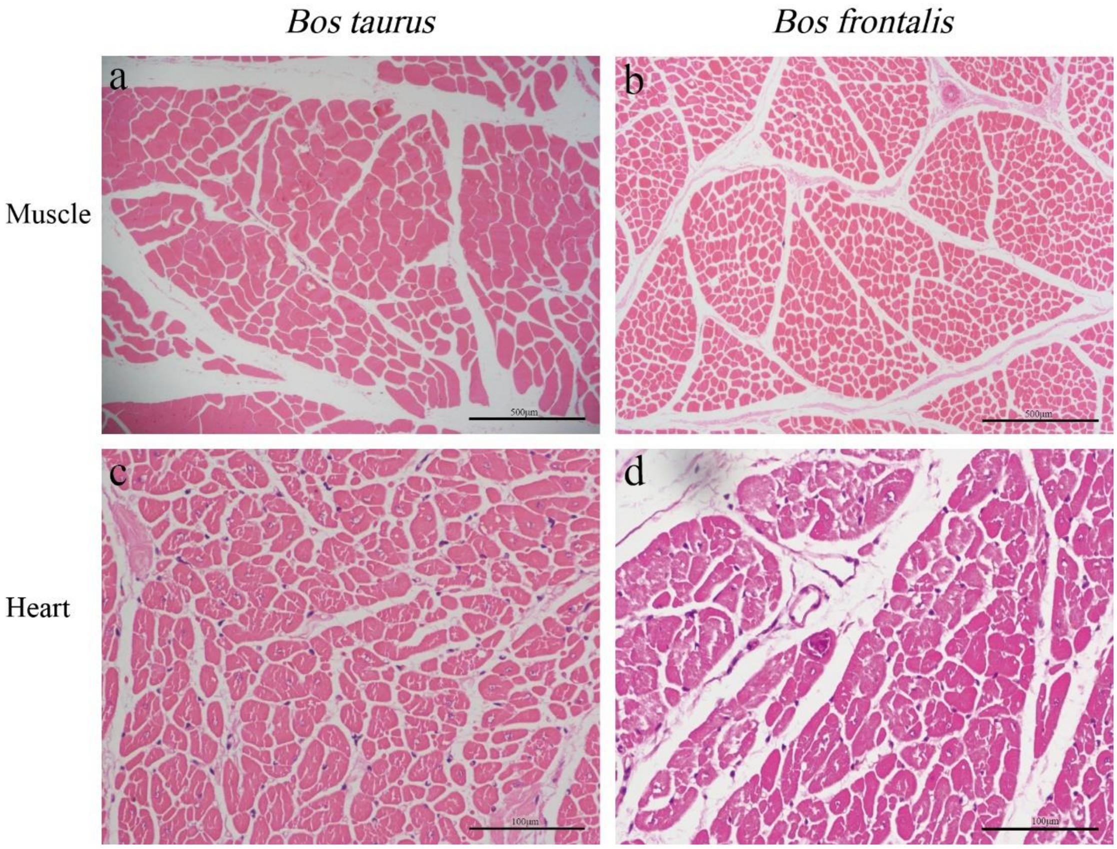

Supplementary Figure 2. Muscle (40×) and heart (200×) cross-section observation in *Bos taurus* and *Bos frontalis* with hematoxylin and eosin (HE) staining.

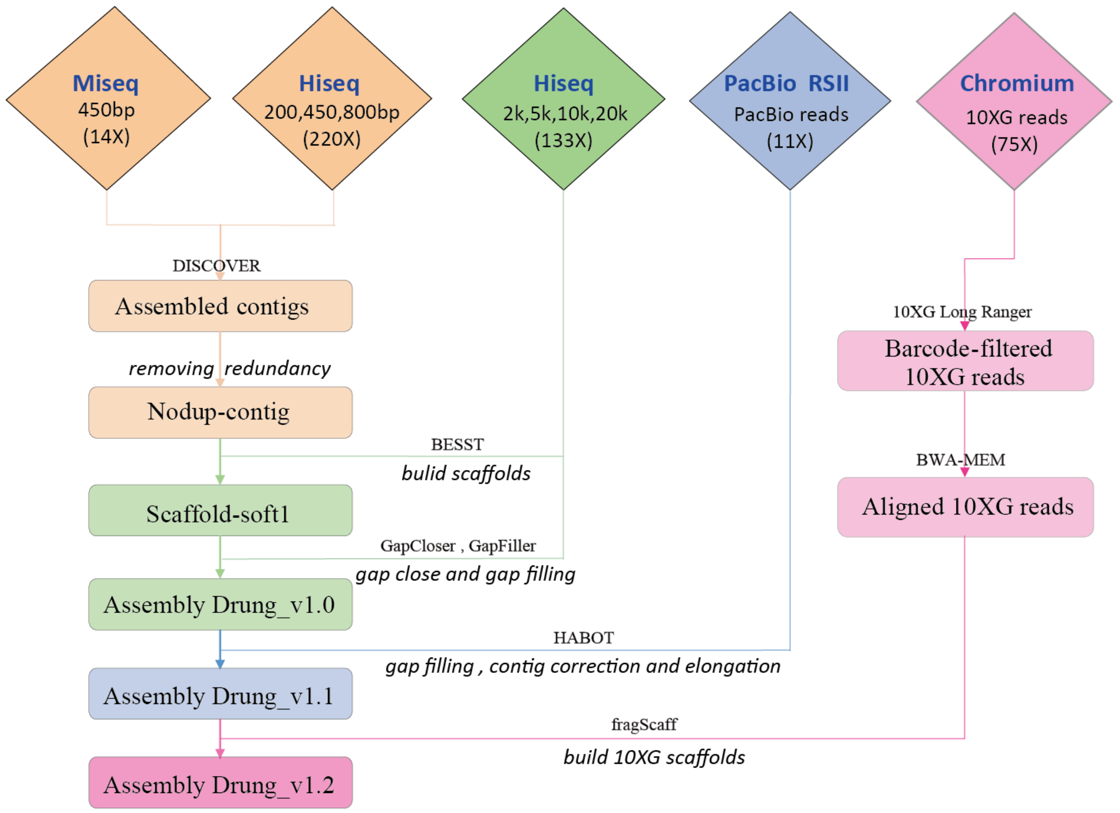

Supplementary Figure 3. Flowchart depicting genome assembly strategy.

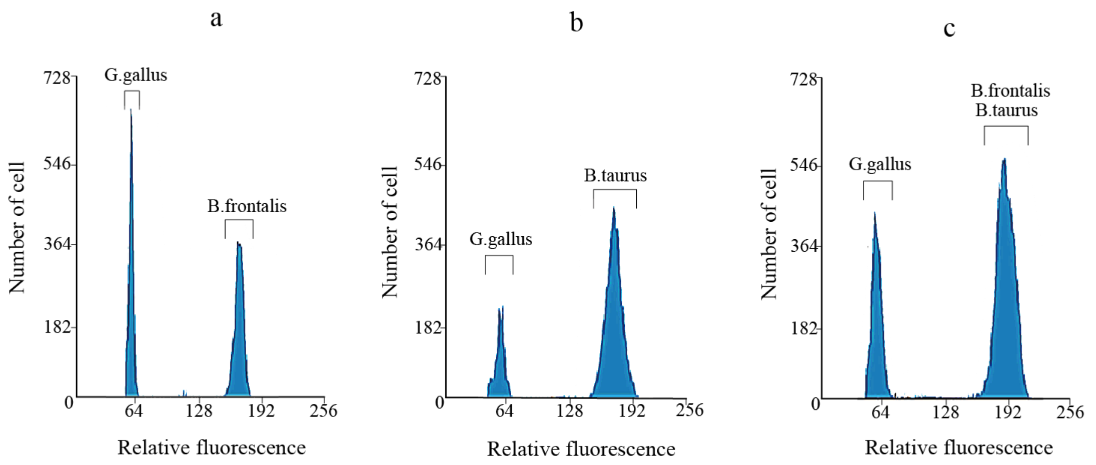

Supplementary Figure 4. The genome size estimation of Drung cattle (*Bos frontalis*) using flow cytometry. The erythrocyte nuclei of chicken (*Gallus gallus*) as an internal reference standard and domestic cattle (*Bos taurus*) as a positive control were selected to determine the genome size of *Bos frontalis*. This illustration shows the flow cytometric histogram of relative DNA content of nuclei from *Bos frontalis* **(a)**, *Bos taurus* **(b)**, and mixtures of *Bos frontalis* and *Bos taurus* **(c)** compared to chicken, respectively. Calculation formula: $C = A1/AH \times H$. Wherein, C indicates the DNA content of the sample to be tested; A1 indicates the fluorescence intensity of the sample to be tested; AH indicates the fluorescence intensity of the reference sample; and H indicates the DNA content of the reference.

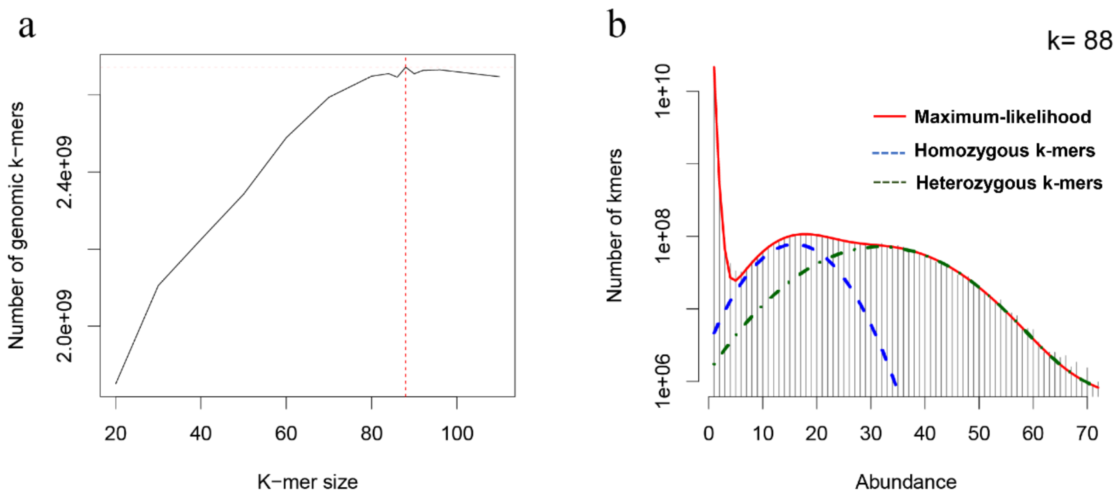

Supplementary Figure 5. k-mer distribution analysis for genome size and heterozygosity estimation of the Drung cattle genome. (a) Estimation of genome size and *k*-mer value using Kmergenie software. **(b)** The abundance histogram and optimized model using a diploid model (*k*= 88). The vertical bar histogram curves are the actual *k*-mer histogram, and the red (solid) curve is the maximum-likelihood fit using our model. In the diploid model graph, the green (dot-dashed) curve models the heterozygous *k*-mers and the blue (dashed) curve models the homozygous *k*-mers.

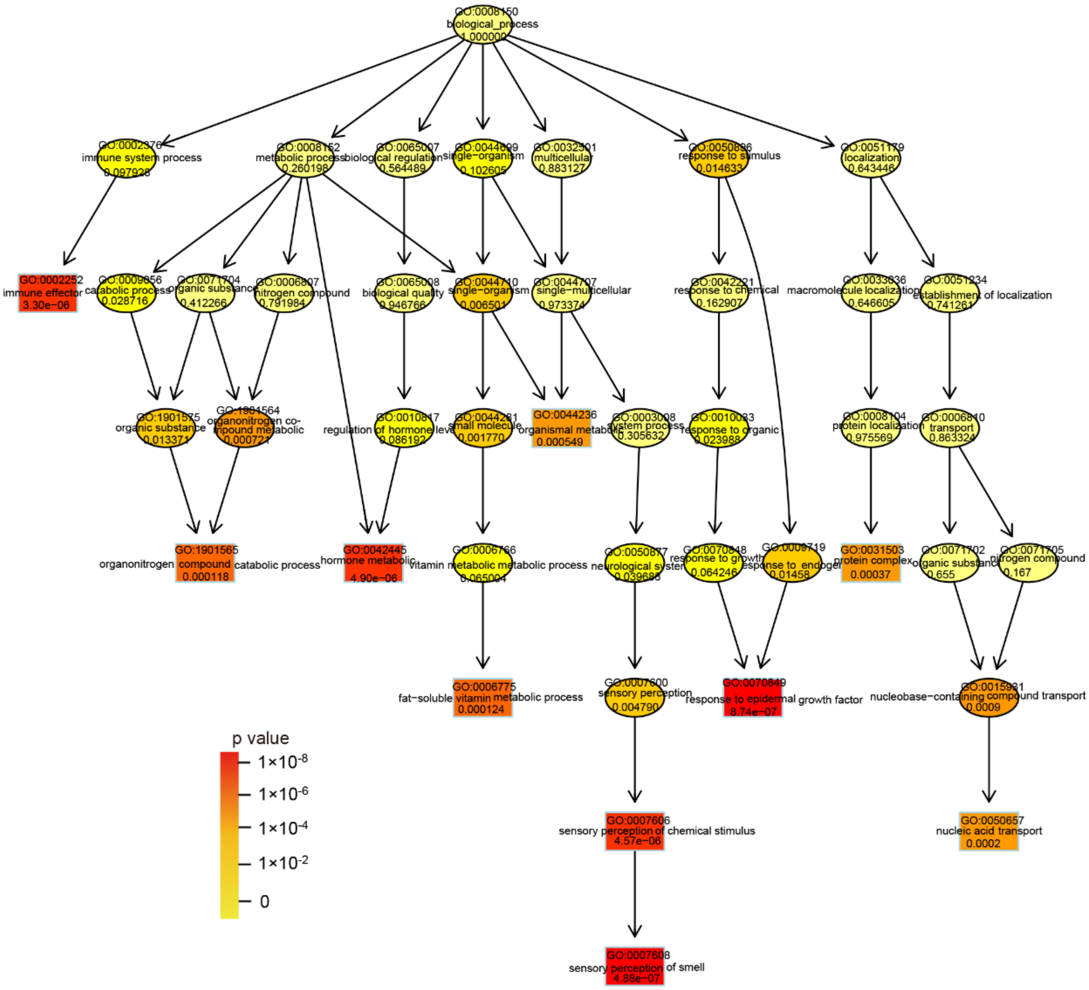

Supplementary Figure 6. GO functional enrichment for segmental duplications (SDs). The color from yellow to red represents the more significant enrichment in a particular GO term.

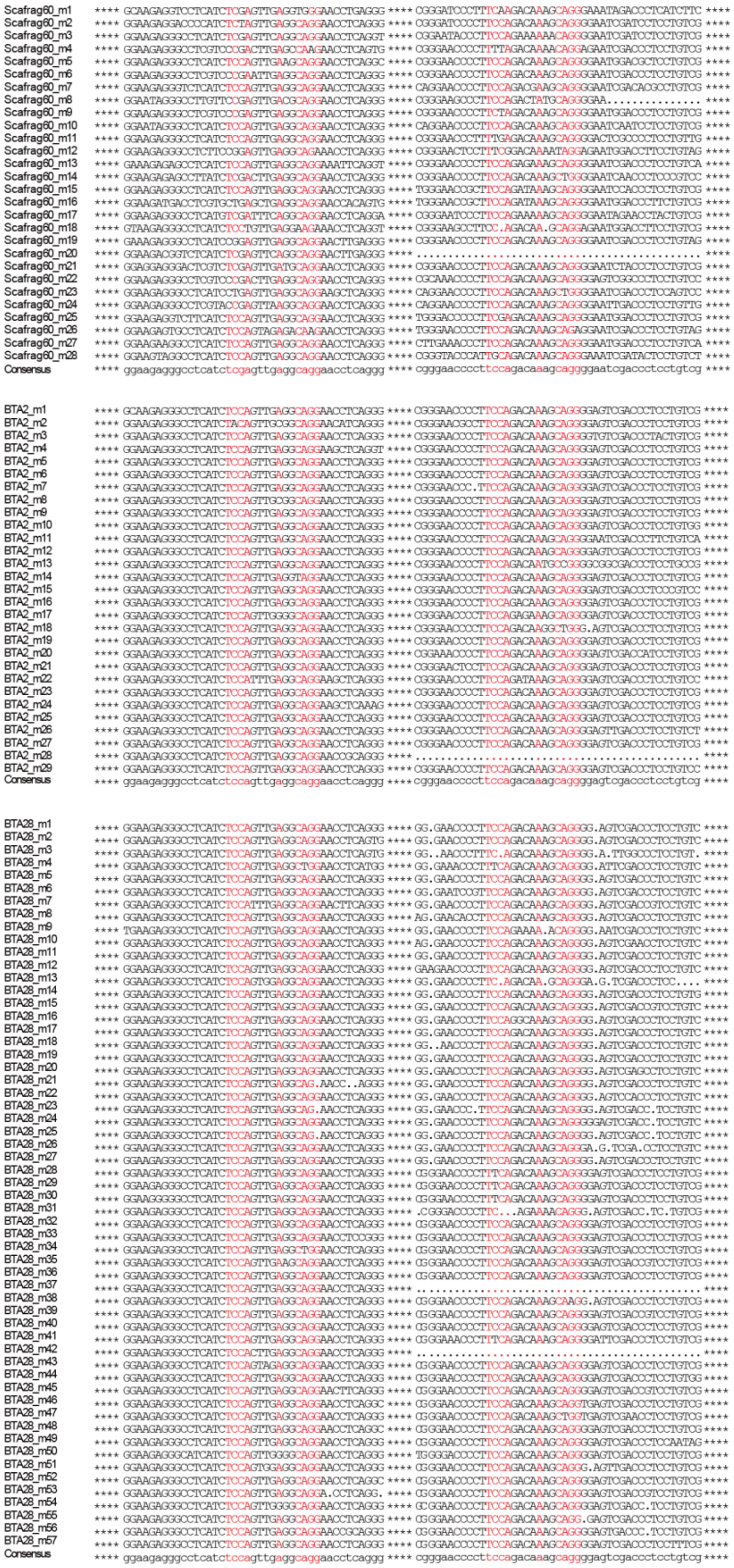

Supplementary Figure 7. Alignment of CENP-B box-like motif in satellite repeat units.

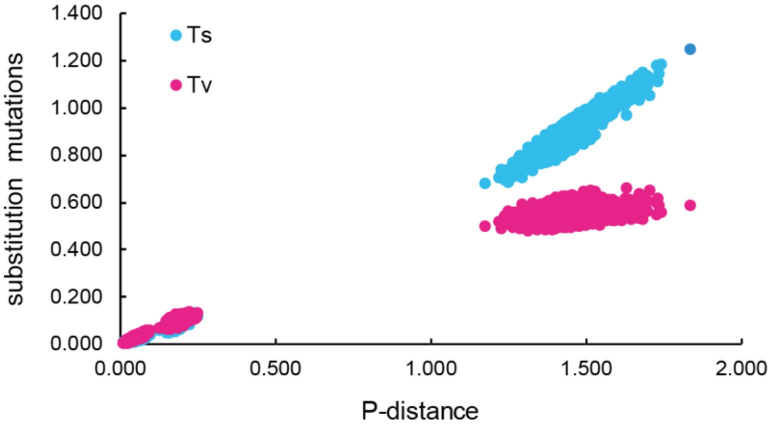

Supplementary Figure 8. The bivariate scatter diagrams of sequence substitution saturation for the satellite repeat units.

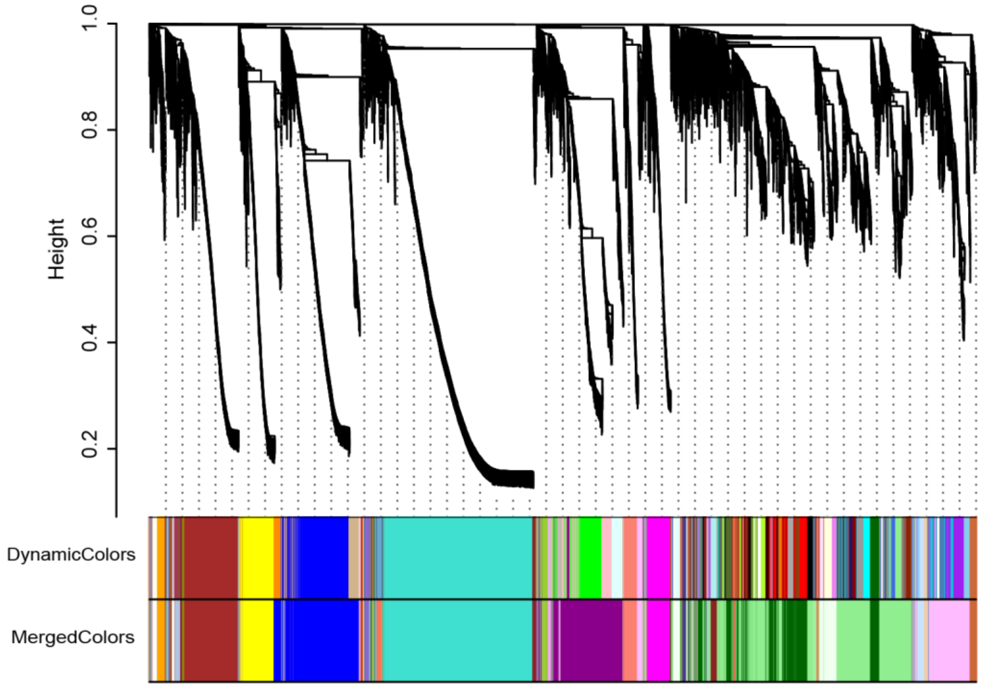

Supplementary Figure 9. Clustering dendrogram for module classification. Genes with similar co-expression patterns were grouped using hierarchical clustering. Co-expressed modules were determined by the dynamic branch cutting methods. Each major branch represents a color-coded module that contain a group of highly connected genes.

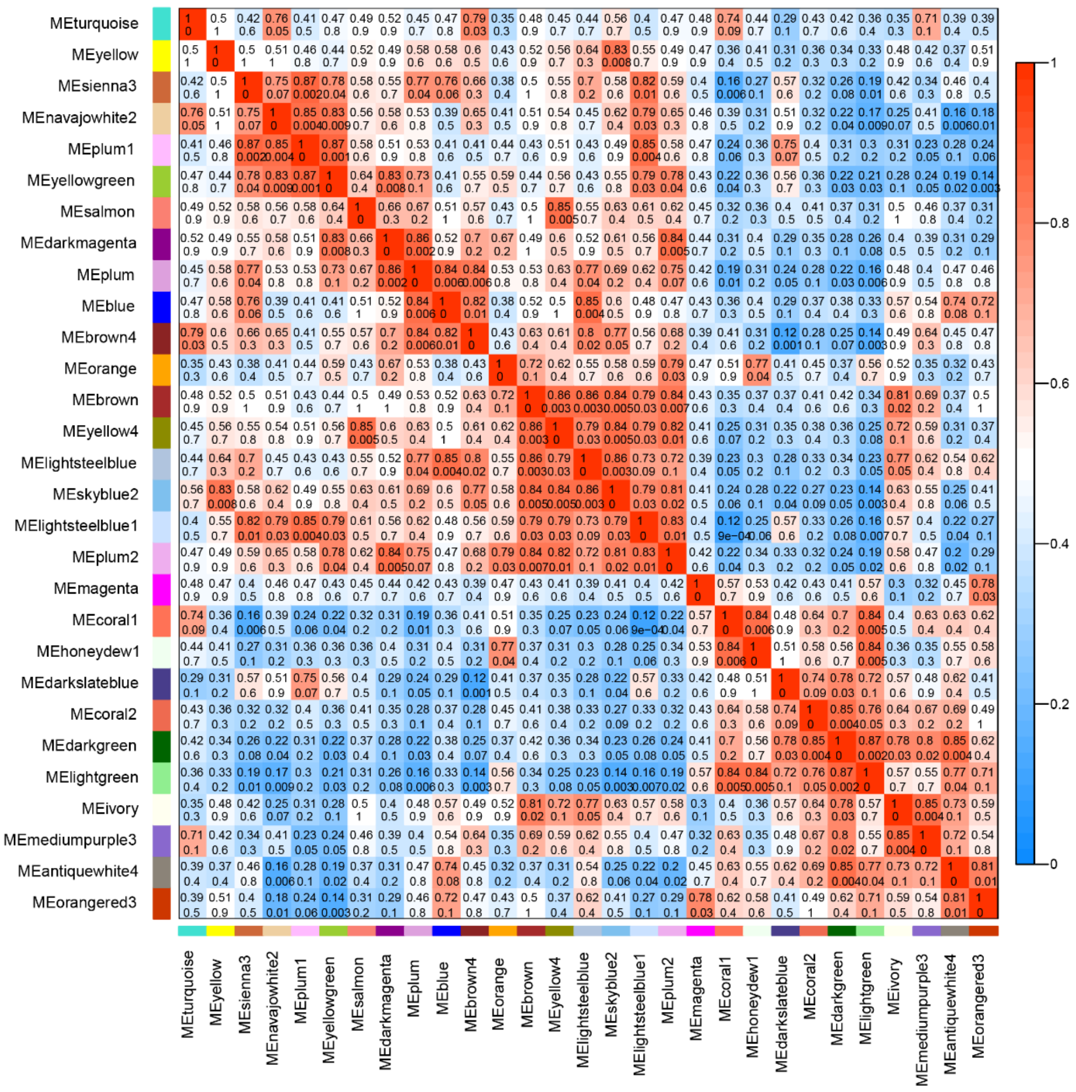

Supplementary Figure 10. Module eigengene adjacency heatmap. Module-eigengenes (ME) in this analysis are defined as the first principal component of a co-expression module matrix. Each row and column correspond to one module eigengene labeled with its corresponding color. The heatmap shows the relatedness of the 29 co-expression modules identified by WGCNA with red being highly related and blue being not related.

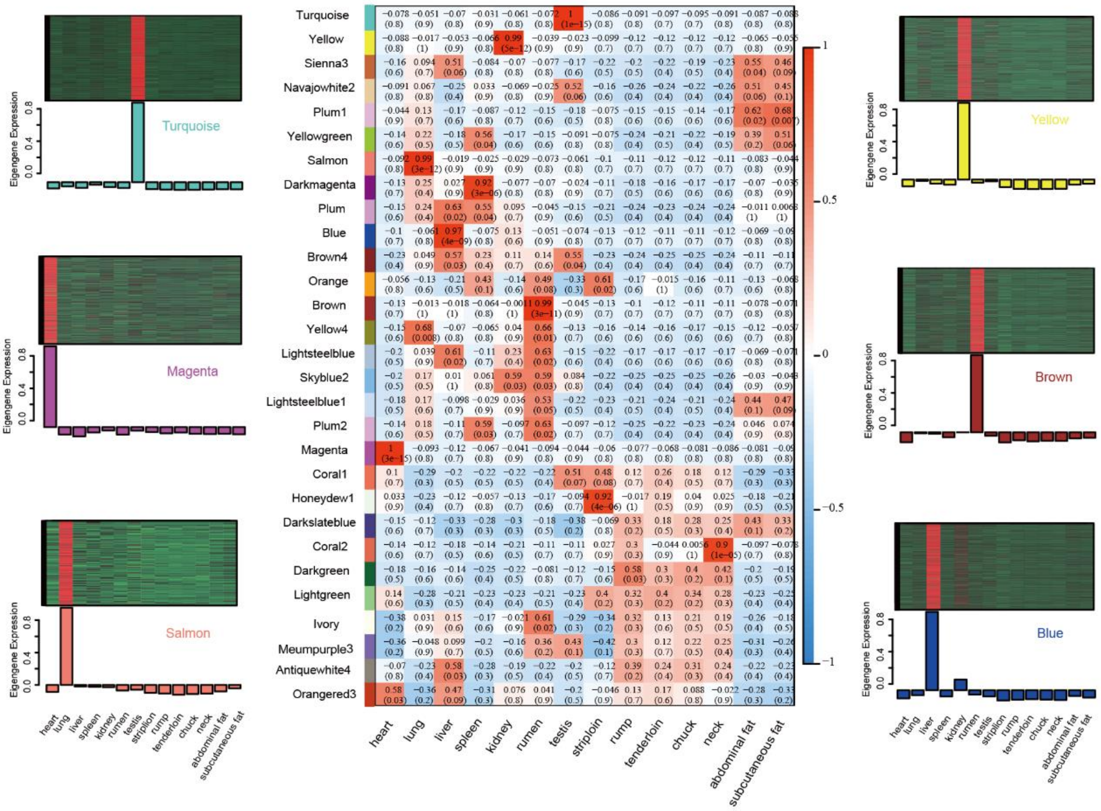

Supplementary Figure 11. Heatmap between 29 modules and 14 tissues. The abscissa axis represents 14 tissues and the ordinate axis represents 29 modules. Pearson correlation coefficients and p values are marked in the boxes. Six of nine tissue-specific modules are shown on both sides of heatmap.

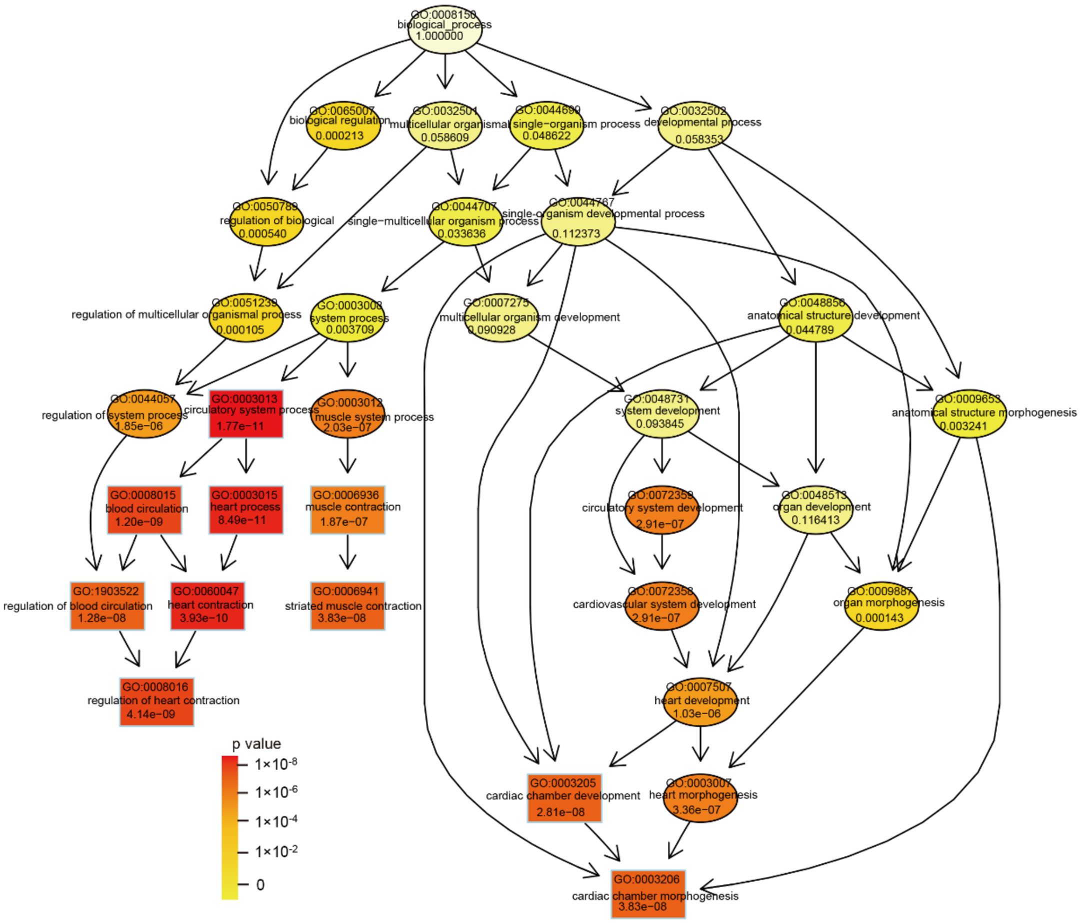

Supplementary Figure 12. GO enrichment analysis for the Magenta-heart module.

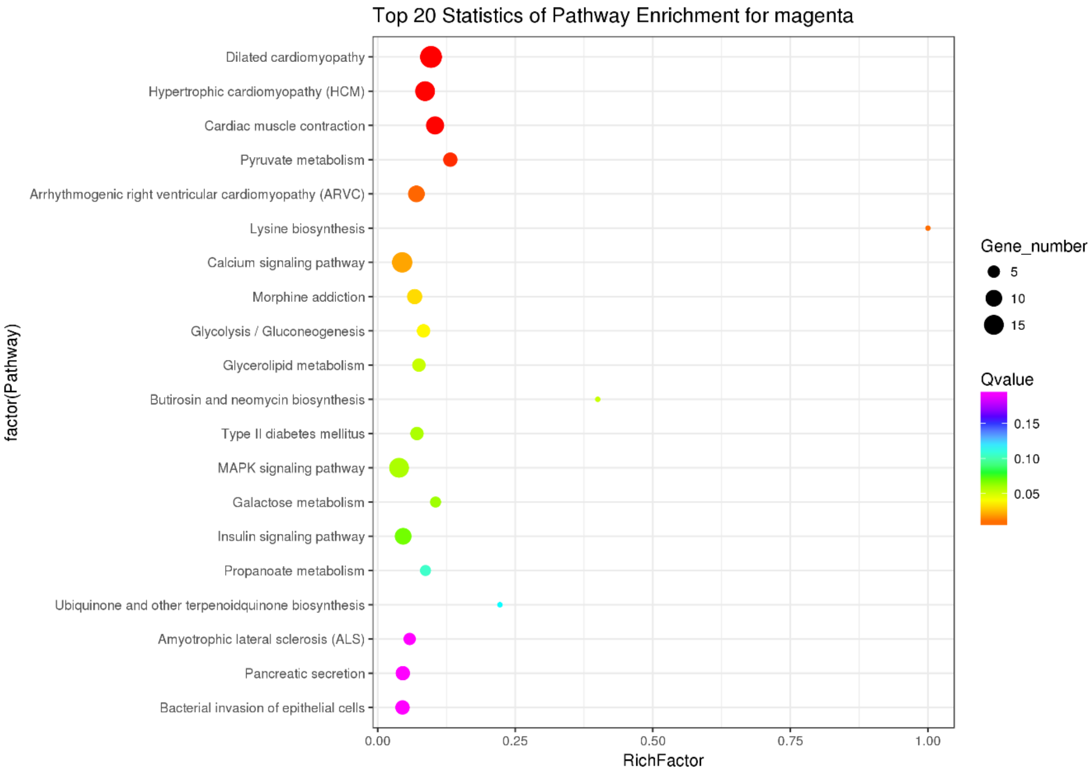

Supplementary Figure 13. Top 20 statistics of KEGG enrichment analysis for the Magenta-heart module.

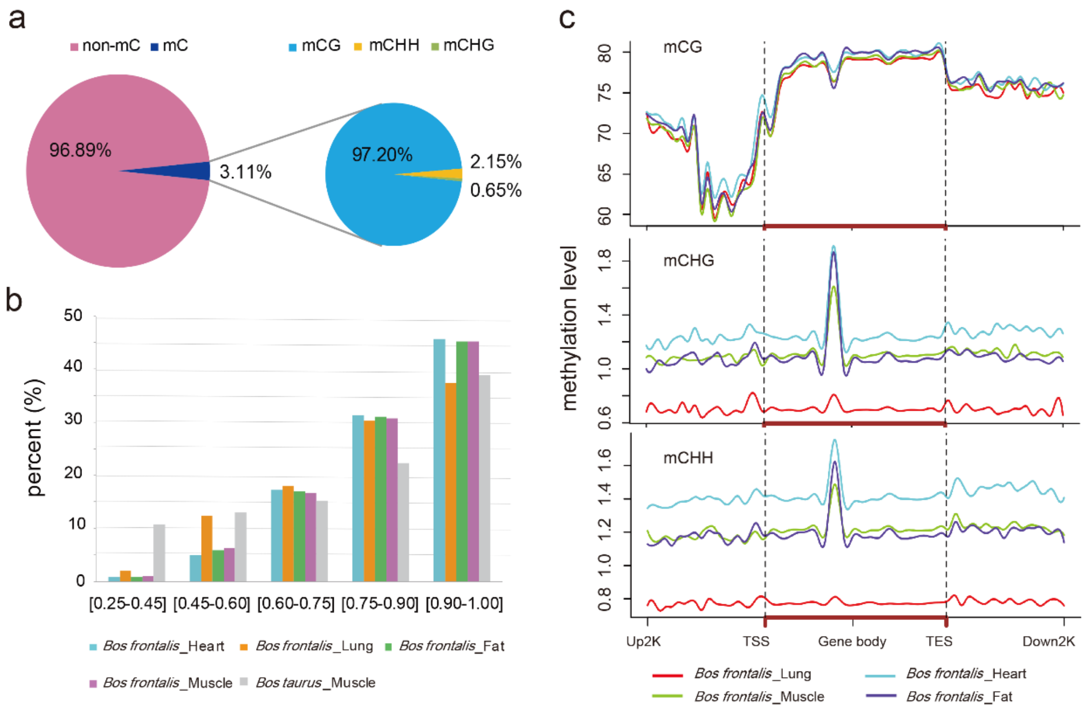

Supplementary Figure 14: Landscape of genome-whole DNA methylation of Drung cattle (*Bos frontalis*). a The DNA methylation percentage of non-mC and mC sites in gayal genome. **b** The distribution of DNA methylation level in tissues. **c** DNA methylation profiles in the gene regions of mCG, mCHG, and mCHH contexts. The gene region was defined as the whole regions that contained 2 kb region upstream of the transcription start site (TSS), gene body from TSS to TES, and 2 kb region downstream of the transcription end site (TES).

Supplementary Tables

Supplementary Table 1. General statistics of repetitive elements in the Drung cattle genome.

Type	Repeat Size (bp)	Rate of genome (%)
TRF	39,855,402	1.46
RepeatMasker	1,106,917,525	40.46
RepeatProteinMask	560,164,743	20.47
De novo	1,060,645,659	38.77
Total	1,229,045,127	44.92

Supplementary Table 2. Functional annotation of predicted genes for Drung cattle (*Bos frontalis*).

		Number	Percentage (%)
Total		20,181	100
Annotated		20,003	99.12
	InterPro	17,448	86.46
	GO	14,453	71.62
	KEGG	15,552	77.06
	SwissProt	19,174	95.01
	TrEMBL	19,990	99.05
Unannotated		178	0.88

Supplementary Table 3. Identification of non-coding RNA genes in the Drung cattle genome.

Type	Copy	Average length(bp)	Total length(bp)	Percentage of genome
miRNA	692	87.17	60,319	0.22%
tRNA	36,201	72.82	2,636,217	9.66%
rRNA				
Total rRNA	1,110	135.49	150,391	0.55%
18S	208	222.08	46,193	0.17%
28S	319	187.83	59,918	0.22%
5.8S	6	63.83	383	0.00%
5S	577	76.08	43,897	0.16%
snRNA				
Total snRNA	1,948	114.32	227.722	0.82%
CD-box	335	89.69	30,047	0.11%
HACA-box	396	134.38	53,214	0.20%
Splicing	1,217	113.92	138,635	0.51%

CD-box: C box (UGAUGA) and the D box (CUGA); HACA-box: H/ACA-type snoRNAs.

Supplementary Table 4. Summary of gene families between *Bos frontalis* in our study and ten other related species.

Species	Genes number	Unclustered genes	Family number	Unique families	Average genes per family
Bos frontalis	20,181	1,017	8,643	143	2.14
Bos taurus	19,951	430	9,626	6	2.03
Bos indicus	18,866	302	9,305	0	2.00
Bos mutus	18,640	160	9,276	1	1.99
Bubalus bubalis	19,592	633	9,536	17	1.99
Bison bison	18,433	392	9,209	4	1.96
Pantholops hodgsonii	23,961	640	9,531	467	2.45
Ovis aries	18,667	388	9,217	9	1.98
Capra hircus	19,854	256	9,591	8	2.04
Ceratotherium simum	19,041	222	9,414	4	2.00
Homo sapiens	22,175	714	9,648	126	2.22

Supplementary Table 5. Statistics of segmental duplications.

Cutoff	# Block	Median size (bp)	Genome coverage (Mb)
>1Kb	41,095	1,563	62.65
>5Kb	1,803	6,280	15.34
>10Kb	190	11,811	37.08
>50Kb	0	0	0

Supplementary Table 6. Identification of functional modules.

Modules	Gene number
antiquewhite4	41
blue	1,335
brown	934
brown4	162
coral1	129
coral2	39
darkgreen	1,094
darkmagenta	1,174
darkslateblue	57
honeydew1	432
ivory	120
lightgreen	2,168
lightsteelblue	132
lightsteelblue1	117
magenta	372
mediumpurple3	84
navajowhite2	50
orange	134
orangered3	34
plum	90
plum1	812
plum2	56
salmon	232
sienna3	88
skyblue2	38
turquoise	2,415
yellow	546
yellow4	37
yellowgreen	88
