# Supplementary material for: A draft genome of Drung cattle reveals clues to its chromosomal fusion and environmental adaptation
Source: Commun Biol. 2022 Apr 13;5:353. doi: 10.1038/s42003-022-03298-9 (PMC9008013; doi:10.1038/s42003-022-03298-9)
Supplement: Supplementary file 3 — Description of Additional Supplementary Files [file 42003_2022_3298_MOESM3_ESM.pdf]

Description of Additional Supplementary Files

File name: Supplementary Data 1
Description: Summary of genome assembly statistics in the Bovinae subfamily.

File name: Supplementary Data 2
Description: Summary of sequencing data for Drung cattle (Bos frontalis) genome assembly.

File name: Supplementary Data 3
Description: The result of assembled statistics of Drung cattle genome in this study.

File name: Supplementary Data 4
Description: Genome size estimation of Drung cattle (Bos frontalis) using C value measurement.

File name: Supplementary Data 5
Description: Assembly completeness evaluation in the Bovinae subfamily using BUSCO.

File name: Supplementary Data 6
Description: Assessment of the transcript coverage with the transcriptome assembly contig (TAC) data.

File name: Supplementary Data 7
Description: Genome-wide methylation levels of four tissues in the Drung cattle genome.

File name: Supplementary Data 8
Description: Classification of transposable elements.

File name: Supplementary Data 9
Description: Statistics of annotated protein-coding genes.

File name: Supplementary Data 10
Description: Statistics of RNA sequencing data for fourteen tissue samples of Drung cattle (Bos frontalis).

File name: Supplementary Data 11
Description: Statistics of gene expression in fourteen tissues of Drung cattle.

File name: Supplementary Data 12
Description: Tissue-specific genes (τ=1) and housekeeping genes (τ)

File name: Supplementary Data 13
Description: Statistics of co-expressed genes in fourteen tissues.

File name: Supplementary Data 14
Description: Unique genes identified in Drung cattle.

File name: Supplementary Data 15
Description: Positively selected genes identified in Drung cattle.

File name: Supplementary Data 16
Description: Functional enrichment analysis of unique genes, positively selected genes, expansion gene families, and contraction gene families in the KEGG database.

File name: Supplementary Data 17
Description: Functional enrichment analysis of segmental duplications (SDs) in the KEGG database.

File name: Supplementary Data 18
Description: Description of gene information in scaffolding 60.

File name: Supplementary Data 19
Description: Analysis of tissue-specific modules and hub genes.

File name: Supplementary Data 20
Description: Hub genes identified in the heart tissue of Drung cattle.

File name: Supplementary Data 21
Description: The evolutionary genes involved in the biological processes associated with circulation system and myocardial contraction of Drung cattle.

File name: Supplementary Data 22
Description: Phylogenetic analysis of the *MYH* gene family in six species of *Bovinae*.

File name: Supplementary Data 23
Description: Functional description of *MYH* gene family in six species of *Bovinae*.
