# Supplementary material for: A draft genome of Drung cattle reveals clues to its chromosomal fusion and environmental adaptation
Source: Commun Biol. 2022 Apr 13;5:353. doi: 10.1038/s42003-022-03298-9 (PMC9008013; doi:10.1038/s42003-022-03298-9)
Supplement: Supplementary file 27 — Reporting Summary [file 42003_2022_3298_MOESM27_ESM.pdf]

nature portfolio

Corresponding author(s): Xue Gao
Last updated by author(s): Mar 13, 2022

Reporting Summary

Nature Portfolio wishes to improve the reproducibility of the work that we publish. This form provides structure for consistency and transparency in reporting. For further information on Nature Portfolio policies, see our Editorial Policies and the Editorial Policy Checklist.

Statistics

For all statistical analyses, confirm that the following items are present in the figure legend, table legend, main text, or Methods section.

n/a	Confirmed	
☐	☒	The exact sample size (*n*) for each experimental group/condition, given as a discrete number and unit of measurement
☐	☒	A statement on whether measurements were taken from distinct samples or whether the same sample was measured repeatedly
☐	☒	The statistical test(s) used AND whether they are one- or two-sided<br>*Only common tests should be described solely by name; describe more complex techniques in the Methods section.*
☒	☐	A description of all covariates tested
☐	☒	A description of any assumptions or corrections, such as tests of normality and adjustment for multiple comparisons
☐	☒	A full description of the statistical parameters including central tendency (e.g. means) or other basic estimates (e.g. regression coefficient) AND variation (e.g. standard deviation) or associated estimates of uncertainty (e.g. confidence intervals)
☐	☒	For null hypothesis testing, the test statistic (e.g. *F*, *t*, *r*) with confidence intervals, effect sizes, degrees of freedom and *P* value noted<br>*Give P values as exact values whenever suitable.*
☒	☐	For Bayesian analysis, information on the choice of priors and Markov chain Monte Carlo settings
☒	☐	For hierarchical and complex designs, identification of the appropriate level for tests and full reporting of outcomes
☒	☐	Estimates of effect sizes (e.g. Cohen's *d*, Pearson's *r*), indicating how they were calculated

Our web collection on statistics for biologists contains articles on many of the points above.

Software and code

Policy information about availability of computer code

Data collection	No commercial/custom code was used for data collection.
Data analysis	Here, we are listing the software URLs of all open-source /commercial tools used for data analysis<br>DISCOVER:https://github.com/leebaird/discover<br>BESST:https://github.com/ksahlin/BESST<br>GapCloser:https://github.com/BGI-Qingdao/TGS-GapCloser<br>GapFiller:https://github.com/robincoxe/GAPfiller_GSM900<br>HABOT2:https://github.com/asarum/HABOT2<br>BWA:https://github.com/lh3/bwa<br>fragScaff:https://sourceforge.net/projects/fragscaff/<br>KmerGenie:http://kmergenie.bx.psu.edu/<br>Bowtie:http://bowtie-bio.sourceforge.net/index.shtml<br>TopHat:https://ccb.jhu.edu/software/tophat/index.shtml<br>Cufflinks:http://cole-trapnell-lab.github.io/cufflinks/<br>Piler: http://www.drive5.com/piler<br>Repeatmasker: http://www.repeatmasker.org<br>Repeatmodeler: http://www.repeatmasker.org/RepeatModeler.html<br>TRF:https://tandem.bu.edu/trf/trf.html<br>LASTZ:https://lastz.github.io/lastz/<br>GlimmerHMM:https://ccb.jhu.edu/software/glimmerhmm/<br>genescan:https://genescan.software.informer.com/3.7/<br>Augustus:http://augustus.gobics.de/<br>TBLASTN:https://blast.ncbi.nlm.nih.gov/

tRNAscan-SE:https://github.com/UCSC-LoweLab/tRNAscan-SE
AxtChain:https://www.rdocumentation.org/packages/CNEr/versions/1.8.3/topics/axtChain
chainPreNet:https://rdocumentation.org/packages/CNEr/versions/1.8.3
Circos:http://mkweb.bcgsc.ca/dev/circos/software/download/
ClustalX:https://clustalx.software.informer.com/2.1/
MEGA:https://en.softonic.com/downloads/mega-for-windows
TreeFam:https://github.com/Ensembl/treefam_tools
PhyML:https://github.com/stephaneguindon/phyml
TimeTree:https://github.com/cran/timetree
CAFÉ:https://hahnlab.github.io/CAFE/download.html
PAML:http://abacus.gene.ucl.ac.uk/software/paml.html
WGCNA:https://horvath.genetics.ucla.edu/html/CoexpressionNetwork/Rpackages/WGCNA/
Cytoscape:https://cytoscape.org/download.html
GeneMANIA:http://genemania.org
Bismark:https://www.bioinformatics.babraham.ac.uk/projects/bismark/

For manuscripts utilizing custom algorithms or software that are central to the research but not yet described in published literature, software must be made available to editors and reviewers. We strongly encourage code deposition in a community repository (e.g. GitHub). See the Nature Portfolio guidelines for submitting code & software for further information.

Data

Policy information about availability of data

All manuscripts must include a data availability statement. This statement should provide the following information, where applicable:

- Accession codes, unique identifiers, or web links for publicly available datasets
- A description of any restrictions on data availability
- For clinical datasets or third party data, please ensure that the statement adheres to our policy

Sequence reads of the Drung cattle genome project has been deposited at the National Genomics Data Center (NGDC) (https://bigd.big.ac.cn/). The accession numbers of sequencing data of the Drung cattle genome assembly using Illumina, PacBio, 10x Genomics platforms is PRJCA004132. The accession numbers of sequencing data of transcriptome and DNA methylation are PRJCA002143 and PRJCA003336, respectively. All other data is available from the corresponding author on reasonable request.

Field-specific reporting

Please select the one below that is the best fit for your research. If you are not sure, read the appropriate sections before making your selection.

☒ Life sciences ☐ Behavioural & social sciences ☐ Ecological, evolutionary & environmental sciences

For a reference copy of the document with all sections, see nature.com/documents/nr-reporting-summary-flat.pdf

Life sciences study design

All studies must disclose on these points even when the disclosure is negative.

Sample size	Blood and tissue samples from an adult male Drung cattle (Bos frontalis) were used for De novo genome sequencing.
Data exclusions	No data were excluded from the analysis.
Replication	Biological replicates were used as required and described in the methods section.
Randomization	Not applicable.
Blinding	Not applicable.

Reporting for specific materials, systems and methods

We require information from authors about some types of materials, experimental systems and methods used in many studies. Here, indicate whether each material, system or method listed is relevant to your study. If you are not sure if a list item applies to your research, read the appropriate section before selecting a response.

Materials & experimental systems

n/a	Involved in the study	
☒	☐	Antibodies
☒	☐	Eukaryotic cell lines
☒	☐	Palaeontology and archaeology
☐	☒	Animals and other organisms
☒	☐	Human research participants
☒	☐	Clinical data
☒	☐	Dual use research of concern

Methods

n/a	Involved in the study	
☒	☐	ChIP-seq
☐	☒	Flow cytometry
☒	☐	MRI-based neuroimaging

Animals and other organisms

Policy information about studies involving animals; ARRIVE guidelines recommended for reporting animal research

Laboratory animals	Not applicable.
Wild animals	Not applicable.
Field-collected samples	Whole blood and tissue samples were collected from adult Drung cattle (Bos frontal) in the Drung Cattle Nature Reserve (-4,000 m altitude) of Drung-Nu Autonomous County of Gongshan, Yunnan province, China
Ethics oversight	Not applicable.

Note that full information on the approval of the study protocol must also be provided in the manuscript.

Flow Cytometry

Plots

Confirm that:

☒ The axis labels state the marker and fluorochrome used (e.g. CD4-FITC).

☒ The axis scales are clearly visible. Include numbers along axes only for bottom left plot of group (a 'group' is an analysis of identical markers).

☐ All plots are contour plots with outliers or pseudocolor plots.

☒ A numerical value for number of cells or percentage (with statistics) is provided.

Methodology

Sample preparation	Here, lymphocyte cells from fresh blood of seven Drung cattle (Bos frontal is) and two Sim mental cattle (Bos taurus) as control were isolated.
Instrument	BD LSRFortesssa (Becton, Dickinson and Company, America)
Software	Summit VS.2.1.1246 software
Cell population abundance	Single-cell suspension concentration was diluted to a final concentration of lOE-6 cells/ml
Gating strategy	Forward and side scatter gating

☒ Tick this box to confirm that a figure exemplifying the gating strategy is provided in the Supplementary Information.
